# The objectives and uses of comparisons in geography textbooks: results of an international comparative analysis

**DOI:** 10.1016/j.heliyon.2020.e04420

**Published:** 2020-08-17

**Authors:** Marine Simon, Alexandra Budke, Frank Schäbitz

**Affiliations:** Institute for Geography Education, University of Cologne, Gronewaldstraße 2, 50931, Cologne, Germany

**Keywords:** Education, Geography education research, Comparison, Comparative method, International textbook research

## Abstract

Comparison is a cognitive process and a method of acquiring geographical knowledge widely promoted within school systems in Germany, France and England. Comparison is an everyday practice, but it is also one of the methods systematically used by geographers and serves various scientific purposes. However, little is known about the functions, extent and objectives of comparison tasks in geography education. This study presents an analysis of 20 textbooks from three countries: Germany, England and France. In this international comparative study we analysed all cognitive tasks involving comparison using qualitative content analysis and descriptive quantitative methods. We developed a reliable classification system for categorising the objectives of comparison tasks. In our results we demonstrated that a large proportion of comparison tasks in textbooks are simple, lower-order tasks. Also, many complex tasks in the three countries involve inductive processes, although we identified some national differences resulting from differing textbook structures. Our system for classifying the objectives of comparison tasks will help develop meaningful tasks, aiming to enhance students’ autonomous and critical thinking, and improve their proficiency in the competencies and methods required in geography education.

## 1 Introduction

Comparison is not only an essential cognitive process, but also facilitates the acquisition and deepening of geographical knowledge. School systems in Germany, France and England widely support the use of comparison in geography education. The three countries included in this study use comparison in three different ways. In France, school curricula at all levels practically systematically include comparisons between cases at various scales (see for example Ministère de l’Education Nationale, 2019, p.14; or Eduscol, 2016, p.2). In Germany, "to compare" is one of the main command verbs used; it is an action that students must carry out at all levels of secondary education in geography classes (DGfG, 2017, p.32). In England, contrasting and differential study of diverse phenomena, cases and countries is an integral part of the geography curriculum (Department for Education and Skills and Qualifications and Curriculum Authority, 2004, p.102). However, none of these three national curricula specify or state the purpose of teaching and learning comparison as a method. Furthermore, there has been little research on this topic in geography education, and existing studies often date back some years (Eichberg, 1972; Stroppe, 1981; Köck and Stonjek, 2005). Hence, comparison is a "black box": a practice that is too obvious to question. Yet, taken as a complex process that must be deeply thought out and justified with cogent argument (Wilcke and Budke, 2019), comparison can serve a range of different cognitive objectives. Teachers and textbook authors must take into account these objectives when designing tasks.

Since the curricula studied provide no precise information about comparison, and given that there has been relatively little research into its purposes in geography education, our research examined textbooks and investigated a variety of comparison tasks, in order to establish the types of tasks set. We analysed 20 textbooks from three countries: Germany (specifically North Rhine-Westphalia and Berlin-Brandenburg), England and France. In this comparative study, we looked at all cognitive tasks in these textbooks that included or implied a comparative process. We developed a reliable framework to categorise the objectives of these comparison tasks. We also undertook qualitative content analysis and quantitative analysis to characterise the different types of tasks in the textbooks studied. Our research questions were:

- How can we categorise the objectives of comparison tasks in geography education and establish a typology?
- To what extent do textbooks from the three countries include comparison tasks, and to what categories do these comparison tasks belong?

The first section of our paper presents the theoretical background and a classification system for comparison tasks. Then we present our methods, which included qualitative and quantitative analysis. The third section presents the results of our analysis of comparison tasks in textbooks, discusses our findings and state the potential of our classification system to help design comparison tasks for use in geography classes.

## 2 Theoretical background: how should comparison tasks be classified?

Comparison is the cognitive act of examining two or more units in conjunction, according to one or more variables, to assess similarities and/or differences (Namy and Gentner, 2002, p.6). In our everyday lives, we use comparison constantly to help us reason and categorise phenomena (Azarian, 2011, p. 115). Comparative analysis is also a scientific method. However, unlike everyday comparison, scientific comparison adopts systematic procedures and techniques in the definition and processing of comparison units and variables (Piovani and Krawczyk, 2017, p.822). The natural, and social sciences also use comparison as a method of acquiring knowledge, building theory and differentiating cases (Lijphart, 1971, p.682; Przeworski and Teune, 1970, p.5; Glaser and Strauss, 1967, p.105; Gervais-Lambony, 1994, p.20). In the social sciences, comparison has been seen as a scientific method for more than a century (Mill, 1882; Durkheim, 1967; Weber, 1921) and has helped to lay the theoretical foundations of the subject area. Comparison was also widely used by early geographers. For example, Humboldt (1807) compared vegetation zones in mountains, and Carl Ritter (1865) compared continents. Geography has a vibrant approach to comparison, especially in urban studies (Nijman, 2015; Robinson, 2006, 2011; Scott and Storper, 2014).

What are the objectives of comparison in the social sciences and geography? The first scientific objective of the comparative method is nomothetic, meaning it is used to determine theories and establish generalisations (Piovani and Krawczyk, 2017, p.823). Thus, it implies adopting a variable-oriented strategy (Ragin and Charles, 1987, pp.54–55) and identifying “universalising explanations” (Tilly, 1984, p.87), theoretical models and even ideal-types (Weber, 1921). The aim is to identify hypotheses and general laws via an inductive approach based on comparing examples (Lijphart, 1971, p.692). Diachronic comparisons can also be used to identify and define processes, via this nomothetic approach (Azarian, 2011, p.116). Some variations may then involve comparing models constructed with other examples in order to confirm or invalidate a pre-existing theory, or to complete or update it, using a deductive approach that involves testing a model with a potentially deviant new case (Lijphart, 1971, p.692; Tilly, 1984, p.116). In geography, for example, the analysis of American cities led to the emergence of urban theories and models that associated the city with modernity, industrialisation, and development (Burgess et al., 1925). These globalising theories have constructed ideal-types such as New York or Chicago, against which other cities have then been compared. This systematic comparison of urban examples with globalising theories is also found in the global city model (Sassen, 1999; Brenner and Keil, 2006).

The second objective of comparison is idiographic (Piovani and Krawczyk, 2017, p.3). This involves a more singular approach to cases and examples (Pickvance, 2001, p.12; Ragin and Charles, 1987, p.54–55), with the goal of demonstrating the uniqueness of observed facts (Gervais-Lambony, 2000, p.10). The nomothetic comparison approach has been criticised for its universal theoretical aims that may reduce the complexity of individual cases, or lose relevance when applied to too many cases (Sartori, 1970). In geography, nomothetic approaches have also been highly criticised for setting up an “imperial methodology” strongly marked by ethnocentrism and neo-colonialism (McFarlane and Robinson, 2012; Robinson, 2006; Roy, 2009). One of the consequences of these approaches is the tendency to identify “other” cities as incomplete in regard to globalised, exemplary models (Peck, 2015, p.161), and the relevance of models or ideal-types in research strongly influenced by Western research structures and theories has been questioned. In response, many authors have called for a theoretical and methodological reconstruction of urban studies, and the establishment of postcolonial and critical research, in order to analyse all cities as “ordinary” (Robinson, 2006, p.109). In a larger sense, these debates raise the question which comparison units to use in geography if we want to avoid perpetuating stereotypical examples and promote decolonising, critical approaches, while also making scientific statements that apply beyond an individual case. Given this, an “intense dialectic between induction and deduction” is always required (Nijman, 2015, p.185).

**Figure 1:**
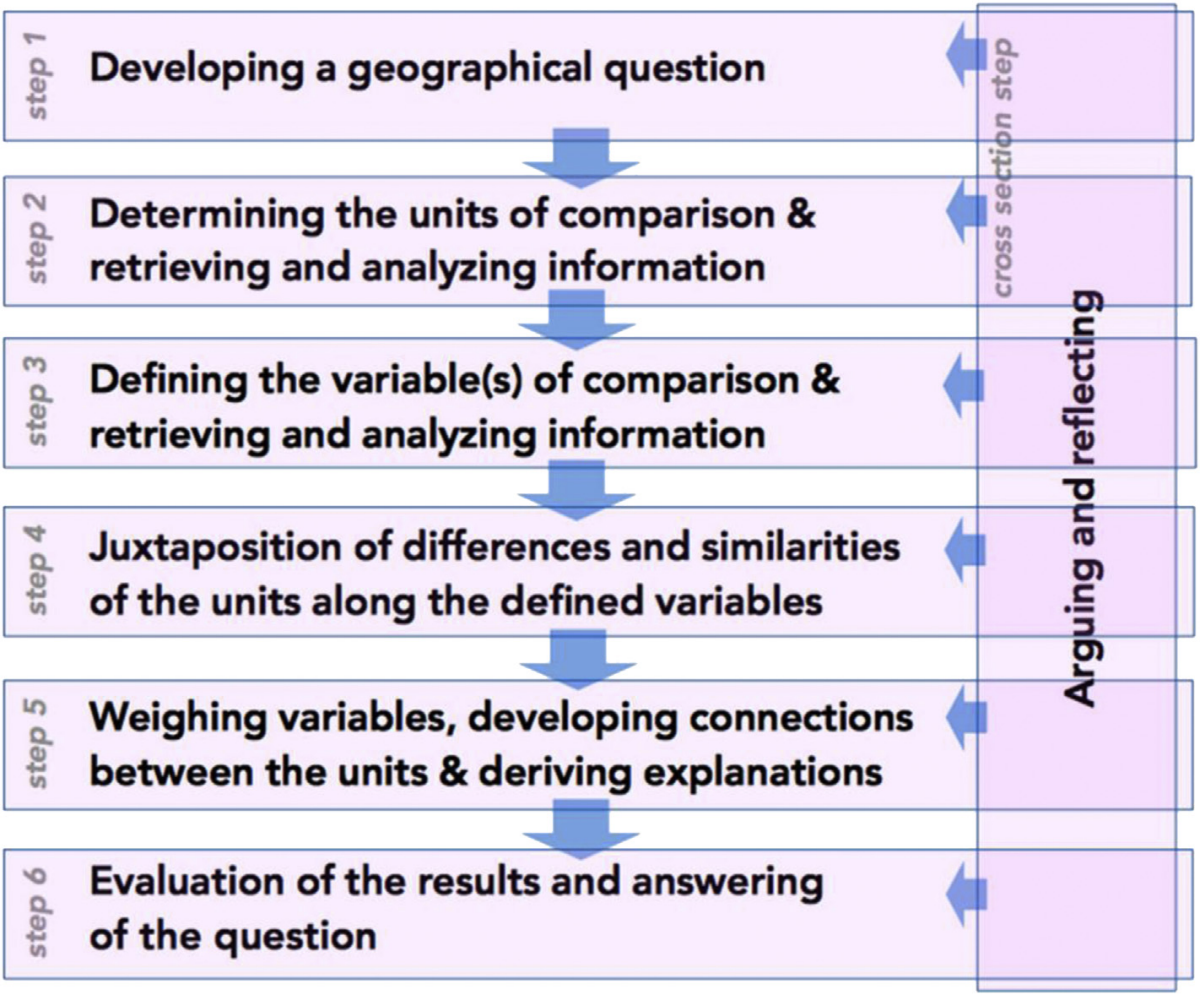
Method of comparison step by step. Wilcke and Budke (2019), p.8.

Little attention has been given to comparison tasks in the research literature on geography education. Contributions to this topic are scarce (Eichberg, 1972; Stroppe, 1981; Köck and Stonjek, 2005). Comparison is often described as having only nomothetic objectives (Köck and Stonjek, 2005, p.258), via an inductive approach (Laske, 2013, p.285). Only Wilcke and Budke (2019) have proposed a six-step model for comparison tasks, in which all steps are carefully thought out and each choice justified with arguments (see Figure 1):

Firstly, the model postulates a preliminary problem that should guide the comparison work. Units of analysis must then be selected (Step 2), followed by variables (Step 3). These choices should be carefully considered and justified with arguments to avoid any risk of ethnocentrism in the definition of the variables and units considered (Piovani and Krawczyk, 2017, p.5). The next step is to contrast the similarities and differences of selected units along identified variables (Step 4). Yet comparing is more than that, since Step 5 involves explaining observed variations and relationships (Pickvance, 2001, p.11). In Step 6, the initial question is answered. This model contributes to defining comparison in geography classes.

The curricula of the three countries studied focus in particular on three different approaches to comparison. Firstly, in France, authors of instructions for teachers and official documentation accompanying curricula, relate comparison to inductive and nomothetic processes (Eduscol, 2016, p.4; Le Mercier, 2010, p.66). Indeed, French and English curricula have both involved in-depth analysis of case studies since the 2000s (Ministère de l’Education Nationale, 2000; Department for Education and Skills and Qualifications and Curriculum Authority, 2004, p.102) and comparison tasks are systematically implemented to compare cases in different locations and of various scales. With this approach, comparison helps to form concepts and rules using an example. Furthermore, teachers are asked to choose “representative” case studies for general geographical processes and concepts (Le Mercier, 2010, p.63). Secondly, in England, geography education has also been influenced by “enquiry-based learning” approaches (Roberts, 2003; Leat, 1998; Ferretti, 2013, p.106). Enquiry-based learning consists of the transposition of a scientific method into the classroom, and is used in higher education (Sonntag et al., 2017). It is also used in the experimental sciences in France during practical work (Reverdy, 2013, p.6). This constructivist approach uses projects, case studies and examples to engage students in solving geographical problems with geographical methods. Thirdly, in Germany, the curricula relate comparison to deductive processes (Senatsverwaltung für Bildung, Jugend und Sport Berlin, 2006, p.14). With fewer case studies and a greater emphasis on models and theory in the curricula, comparison tasks aim to train students to use models or to validate theory. There is therefore, stark contrast between the broad adoption of comparison in curricula, textbooks and the scientific context, and the lack of reflection on its precise objectives in geography education research.

Therefore, classifying comparison tasks into their inductive, deductive or idiographic purposes seems necessary to develop meaningful tasks in a competency-oriented geography class. Here we propose a classification system for comparison tasks according to their educational objectives in geography classes (see Table 1). This typology emerged while studying the objectives of comparison tasks in textbooks via a qualitative approach. Indeed, the analysis of the tasks’ objectives showed that not only did they correspond to the various aforementioned scientific comparison objectives, but could also be divided into higher-order and lower-order tasks (Krathwohl, 2002; Jo and Bednarz, 2009; Krause et al., 2017).

**Table 1:** Classification of comparison task objectives. Own elaboration.

	Simple comparison tasks	Complex comparison tasks
Type 1: Comparisons to build theory or to differentiate case studies	Type 1.1	Type 1.2.1
	Comparison of two or more units to establish if they are similar or different via a simple juxtaposition. *e.g.: “Compare the flooding caused by a tsunami (see pp. 40–41), the flooding caused by flood events in Bangladesh (see pp. 46–49) and the flooding caused by flood events in Germany in terms of their causes, duration and consequences. Find similarities and differences.” (*Hellmanns et al., 2016*, p. 51)*	Comparison of two or more units, including explanation of the findings, in order to establish a general rule, a concept, a model or a definition in an inductive way. => NOMOTHETIC APPROACH *e.g.: “Compare the altitude levels of the mountains near the Equator (Andes) with those of the mountains far from the Equator (Alps). Make a list of similarities and differences. Formulate a rule that describes the changes in altitude levels from the equator to the pole.” (*Bette et al., 2017*, p. 153)*
		Type 1.2.2
		Comparison of two or more units, including explanation of the findings, in order to better understand examples in an interpretive way. => IDIOGRAPHIC APPROACH *e.g. “Thinking through your enquiry: You have been asked to write a report on behalf of the European Commission to explain why more industry and jobs are needed in the South of Italy. (…) You should write your report in five sections: 1. Introduction - How the South is different from the North.” (*Hillary et al., 2001*, p. 89)*
Type 2: Comparisons to apply or to test models	Type 2.1	Type 2.2
	Comparison of two or more units in order to apply a model or an ideal type, or to use a general rule, concept, model or definition. *e.g.: “Compare the model [of demographic transition] with reality: describe similarities and differences.” (*Bette et al., 2018*, p. 51)*	Comparison of two or more units to reflect on or criticise a model or an ideal type, or criticise a general rule, concept, model or definition. *e.g: “Model application. A. Work out the functional structure of Berlin on the basis of a large city map (e.g. in the Atlas). B) Assign it to a model of the functional outline. C) Then assess the significance of these models.” (*Boeti et al., 2015*, p. 331)*
Type 3: Comparisons to rank	Type 3.1	Type 3.2
	Ranking task for more than two examples to order them on a scale. *e.g: “Which EU countries are the most developed? The least developed?” (*Plaza et al., 2016a, Plaza et al., 2016b, Plaza et al., 2016c, Plaza et al., 2016d*, p. 369)*	Ranking task with explanation and reflection on the variables, with a quantitative or hierarchical dimension, to define types or a typology. *e.g: “Name the months in which there is the smallest and largest selection of fruit and vegetables from Germany. Explain.”* (Bette et. al., 2016, *p. 149)*
Type 4: Temporal comparisons	Type 4.1	Type 4.2
	Temporal comparison to characterise whether a change or a pattern are observed in time. *e.g: “Describe how agriculture changed between 1950 and 2013 using photos 1 and*Table 2*.” (*Bette et. al., 2016*, p. 152)*	Temporal comparison to characterise whether a change or a pattern is observed over time in order to provide an explanation and reflection on processes or consistencies. *e.g: “What inequalities between areas have become more severe as a result of metropolisation?” (*Janin et al., 2019a, Janin et al., 2019b*, p. 37)*
Type 5: Comparisons to promote media literacy	Type 5.1	Type 5.2
	Comparison of different types of documents or sources, to learn how to read them. *e.g: “Compare the aerial photos of Münster: What exactly can you see, what less clearly?” (*Bette et. al., 2016*, p. 25)*	Comparison of different types of documents and sources in relation to the content or to the goals of the documents, to reflect on their use. *e.g: “What sort of maps would be most appropriate for displaying the following? - the population distribution within a country - the location of dairy farms in the UK - the ethnicity of different cities in the UK - the number of doctors per 1000 of the population of the UK - the main global migration of the past 30 years. Justify your choices” (*Plaza et al., 2016a, Plaza et al., 2016b, Plaza et al., 2016c, Plaza et al., 2016d*, p. 629).*

The distinction between simple and complex tasks (see Table 1) corresponds, firstly, to the division stated by P. Gervais-Lambony (2000) and A. Lijphart (1971). They define simple comparison as everyday comparison, or as the juxtaposition of similarities and differences, without reflection or scientific objective. On the contrary, according to these authors, complex comparison implies an analysis and serves idiographic or nomothetic purposes. Second, this differentiation also corresponds to a division between higher-order or lower-order tasks based on existing taxonomies of educational objectives (Krathwohl, 2002; Jo and Bednarz, 2009; Krause et al., 2017). In addition to differentiating between simple and complex comparison goals, we identified, through category building, another five subtypes related to the nature of the comparison goals. Type 1 involves a nomothetic or interpretive approach, which is based on comparison between examples or case studies, and whether the task aims to generalise and establish rules, define concepts or properties, construct a model or develop a definition using hypothesis and theorisation (Skocpol and Somers, 1980; Lijphart, 1971), or aims to better understand and differentiate case studies. In its simplest version, it is limited to juxtaposing examples without drawing any conclusions or making any generalities. Type 2 consists of the reverse approach, which is deductive; the comparison consists of applying a model to a case study in order to practice or repeat the model. The complex version of this type involves confirming, criticising or refining the model through comparison, particularly through “deviant” case studies (Lijphart, 1971, p.692; Weber, 1921, p.1). The objective in Type 3 is to build a typology or classification system based on the comparison of several examples. In its simple form it is simply a matter of ordering the units being compared, whilst in its more complex form this comparison leads to defining types using precisely chosen variables in a reflective process (Scott and Storper., 2014, p.3). Type 4 involves a temporal dimension that includes comparing units over time to distinguish or conceptualise processes or consistencies (Bartolini, 1993, p.136). A simple form of this comparison is comparing the elements over time without questioning the factors or processes underlying the observed changes. Finally, a specific category, Type 5, consists of the comparison and analysis of documents and sources by students. Here, the aim of comparison is to acquire skills in media literacy, or simply to identify different types of media. This category, which is broadly visible in our studied textbooks, distances itself from the others in that it considers the media used in the comparison in its own right. We used this five-type classification system to analyse tasks in our examination of textbooks.

## 3 Methodology

To understand the use of comparison we undertook an analysis of comparison tasks in 20 textbooks from France, England, and the German states of North Rhine-Westphalia and Berlin-Brandenburg.

Analysing textbooks from three countries requires an analysis of the context of geography education in each country to avoid the biases inherent in any comparative research. A common aspect of the use of comparison in the three countries is that, since the 1990's, they have all adopted an approach focused on skills and competencies, following a general trend across education systems in Europe and more broadly (Young et al., 2014; Klieme et al., 2007; Koch and Laske, 2014; Thémines, 2016). The focus shift from knowledge to competencies in the three countries is reflected in the curricula, which are now more output-oriented than input- or knowledge-oriented. But there are differences in the status of geography as a school subject and in the progressive specialisation of students, which occurs at different rates in different school systems. We could not include elementary schools since German curricula for these levels do not distinguish geography as an independent subject. Whilst in Germany, as in France, geography can be the subject of specialised study or more intensive teaching from 16-18 years of age while remaining part of the common basic education, in England, geography teaching is only compulsory until the age of 14 and is then taught as an elective subject. Other differences are also apparent: firstly, while French and German secondary school curricula are very precise (about 30 pages depending on the level), English curricula are not very detailed (6–10 pages). Secondly, France has another particularity: geography is always taught in close relationship to history with the two disciplines considered to be sister disciplines (Tutiaux-Guillon and Nicole, 2008), both courses are taught by the same teacher and they are sometimes required to echo each other. As a consequence, for our study we only selected comparative tasks from the geography section of the French textbooks.

Our selection included textbooks from five different series and three other textbooks intended for students of secondary schools between 10 and 16 + years of age. The textbook series selected were, for Germany, *Terra, 1st ed.* (Bette et al., 2016, 2017, 2018; Boeti et al., 2015) and *Seydlitz Geografie* (Amstfeld et al., 2012; Hellmanns et al., 2016; Fleischfresser et al., 2016; Felsch et al., 2011); for France, we selected the textbook series, first, from Hachette *Histoire-géographie-EMC* (Plaza et al., 2016a, Plaza et al., 2016b, Plaza et al., 2016c, Plaza et al., 2016d), and second, from Nathan *Géographie* (Janin et al., 2019a, Janin et al., 2019b) supplemented with *Géographie Terminale* (Janin, 2016); for England, we chose *Think through geography, 6th ed.* (Hillary et al., 2000, 2001, 2002) and completed the selection with two textbooks for older students, *AQA GCSE (9-1) – Geography* (Widdowson et al., 2016) and *AQA A-Level - Geography, 4th ed.* (Skinner et al., 2016). The books came from publishers whose titles are commonly used in schools in the three countries. We also selected the textbooks based on their publishing dates. For France, we used the latest editions, and the German textbooks were also quite recent. For England, though, we deliberately chose three textbooks from an older curriculum implementing enquiry-based learning approaches. These constructivist approaches (Roberts, 2010, p.6; Ferretti, 2013, p.106) were used in English curricula from the 2000s and aimed to develop students’ scientific methods. The inclusion of textbooks applying enquiry-based approaches allowed us to investigate a possible English exception in the treatment of comparison within the tasks. These older textbooks were also supplemented with recent English textbooks corresponding to upper secondary school level.

Textbooks can not only be defined as pure educational media, but also as socio-cultural media representing a self-image of society that is, in many ways, filtered, pre-structured and controlled (Höhne, 2003, p.73–74). They are essential in the preparation and implementation of teaching sequences (Matthes and Schütze, 2011, p.9) and sometimes replace, or interpret, the curriculum. For Lee and Catling (2017, p.345), textbooks are the result of programmatic orientation and curricula, and differ depending on whether education systems promote closed or open teaching approaches. While in a closed, content-oriented approach, textbooks "emphasize mastery of key concepts and principles", in an open or student-oriented system, textbooks allow students to develop their knowledge more independently. The studied books presented differences in structure that reflect differences in curricula. In Germany the books were divided into thematic sections, the French textbooks were structured according to thematic-scale, with numerous case studies, and in England the structure was determined by the didactic "enquiry". Nonetheless, the studied books also had common features, all offering thematic double-pages, for example.

Tasks in textbooks are seen as vital for students to become competent in a skill (Matthes and Schütze, 2011, p.10, Menck, 2011, p.24). The analysis undertaken in this study considers the place of tasks in the "textbook system" (Niclot, 2001, p.103). Indeed, tasks are not only an instruction or a sentence, but are part of a production context, and are part of the page, double-page, or chapter to which they relate. While in Germany and England the words "task" and “*Aufgabe*” have a similar meaning, in France the word “*tâche*" is used for the so-called "*tâches complexes*”, which originate from language education and involve several stages and a final result produced by the student. Other types of tasks are called "*questions*" or "*exercices*" formulated using an "instruction"^1^ or sometimes "evaluation”. Tasks fulfil different functions and requirements depending on their purpose in the chapter, and thus also correspond to different competencies such as memorising, understanding, applying, analysing, creating and evaluating (Bloom, 1956; Krathwohl, 2002). In curricula that are increasingly competency-based and output-oriented, tasks are critical to the acquisition and exercise of these skills.

In the 20 textbooks studied, we identified 10,681 tasks, which were located in different chapter sections and fulfilled different functions. Across textbooks from the three countries, tasks were often visibly distinct from the textual sections aimed at transmitting knowledge (which we named “Main lesson”) and were often associated with documents that the students had to study. In textbook sections involving case studies, tasks and documents were the main elements of the double-page spreads (we identified these sections as “Case studies”). Textbooks from all three countries often included sections designed to help students practice methodological skills, we therefore also included tasks with this purpose (which we refer to as “Methodology”). These sections were usually located at the end of chapters, as were “Revision” sections which were often limited to reproducing information or verifying knowledge acquisition. Tasks were either one isolated task, or included a set of subtasks. In the latter case, the sets of tasks often followed a pattern in which students had first to select or reproduce information, then to apply or explain it, and finally assess it. This corresponds to the three hierarchical steps identified in taxonomies of educational objectives (Bloom, 1956; Krathwohl, 2002) and widely promoted in the German school system (e.g. DGfG, 2017, p. 31–32).

From this range of 10,681 tasks, all exercises involving a comparison task were selected and counted. Comparison tasks were defined as those that consisted of one or more subtasks that engaged students in the production or reception of a comparison, while contrasting the units of comparison according to one or more variables. After applying this definition to the 20 textbooks studied, 981 comparison tasks were selected for analysis (9.18% of the total number of tasks). The following sections present how we processed this data, firstly through a qualitative analysis, and secondly, through quantitative analysis.

We built and refined our classification of tasks into different types (see Table 1) using inductive-deductive category construction (Mayring, 2015 [1982], p.61) through a qualitative analysis. The classification was verified and refined using the Kappa coefficient calculation (Janko and Knecht, 2014): three trained judges were asked to classify all the tasks into the eleven categories formed by the types in their simple and complex form. We obtained the final Kappa coefficient of 0.66, which can be characterised as substantial (Landis and Koch, 1977, p. 165). This allowed us to assess the inter-rater reliability and the clarity of the classification, which was then used via a quantitative analysis to study the goals and the requirements of all comparison tasks from the textbooks for each of the three countries.

Along with identification variables (country, age of students), we classified the tasks in relation to specific variables concerning the tasks’ level of complexity (see Table 1). The following variables were then used (see Table 2):

**Table 2:** List of variables used in the textbook analysis.

Elements analysed	Variables	Levels
General aspects	Country	Germany, England, France
	Age of the students	10-11, 12–13, 14–15, 16+
	Location of the task in the chapter	Lesson, case study, methodology, revision
Comparison types and objectives (as presented in Section 1.1)	Presence of scalar comparison	Presence/absence of scalar dimension
	Comparison types	Type 1.1 to Type 5.2 (see Table 1)

The first three variables allowed us to differentiate tasks according to country and targeted age group, and to identify the extent to which comparison tasks were integrated into the textbooks. The second group of variables enabled us to study the objectives of comparison tasks in textbooks. Here we studied whether tasks included comparison between units with different scalar dimensions, and identified the objectives of tasks using our typology (see Table 1). We differentiated between complex and simple tasks by taking into account what was explicitly asked of students. The following task is an example: “Compare the selected countries according to their ecological footprint” (Boeti et al., 2015, p. 257), which is from a German book for older students (category 16+ in the variable “Age of the students”), and is located in the main lesson part of the chapter on agriculture in different climatic zones (Location of the task in the chapter). The task is not formulated in a way that explicitly asks to interpret or explain the results, although it would be possible for students to do so. This task is therefore a simple ranking task (Type 3.1 in the comparison objectives, see Table 1). The comparison does not entail a change of focus in the given scale (Presence of scalar comparison).

After classifying the comparison tasks via the variables presented above, we carried out a descriptive statistical analysis of frequencies. These were cross-tabulated by country and age to undertake a comparison. As our sample exceeded a size of 100 and individual counts were not inferior to one, we also applied the Pearson Chi-Square Test to test independence between variables, using the commonly accepted level of confidence of 0.05. We complemented our quantitative analysis with different examples from tasks, studied in the textbook context, to better understand and interpret our results. In the following section, we present the results obtained from these analyses.

## 4 Results and discussion

We identified that 9.18% of all tasks in the textbooks studied were comparison tasks, which is a significant number. This indicates that comparative tasks in the context of textbooks in all three countries have important educational functions and are tasks that students often have to perform. However, there were considerable differences between countries; while in Germany comparison tasks represented 9.72% of tasks, we found there were fewer comparison tasks in France (6.25%) and more in England (11.72%), in proportion to the overall percentage. There were also differences in the proportion of comparison tasks within overall tasks, depending on student age: in textbooks intended for 10-11 year-old students, 8.7% of tasks were comparison tasks, whereas the percentage was 7.3% in textbooks for 12-13 year-old students, 9.7% in textbooks for 14-15 year-old students, and 10.5% in textbooks for students aged 16 and over. This demonstrated that the number of comparison tasks increased slightly with the age of students.

### 4.1 A general polarised distribution of comparison types

To better understand the functions and purposes of comparison tasks in the textbooks for our three chosen countries, all comparison tasks were classified into the different types of comparison previously identified (see Table 1).

Overall results showed that 50.15% of the comparison tasks analysed were simple tasks and 49.85% were complex tasks, meaning that more than half the tasks lacked higher-order goals, and consequently could not facilitate the acquisition of meaningful competencies or geographical skills. Type 1, in simple and complex versions, represented 61.36% of all tasks. Although this is in line with some of the main objectives of comparison in geography (Peck, 2015; Robinson, 2011), there is little use of the possible variations between comparison types, which could serve different objectives.

**Figure 2:**
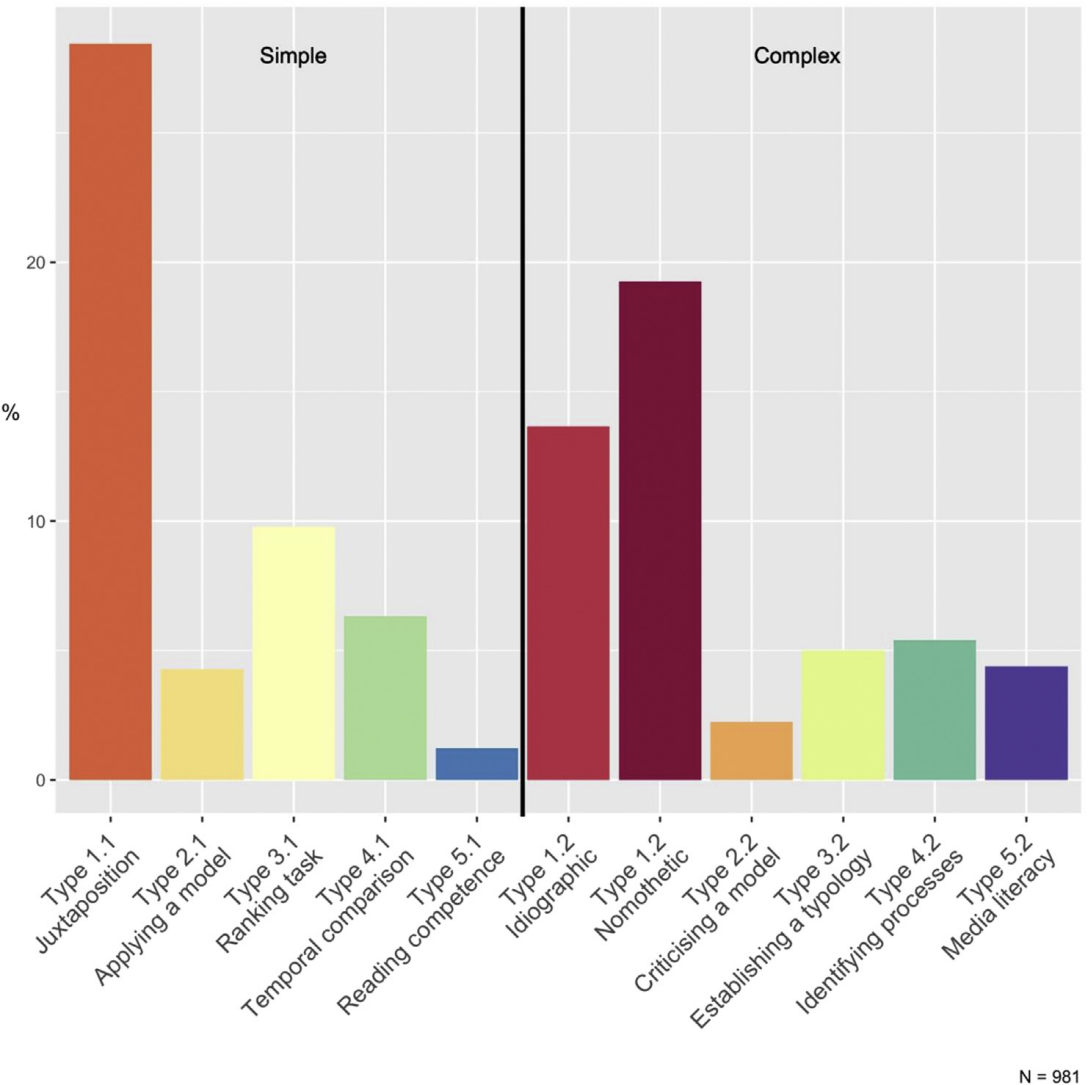
Distribution of comparison task goals. Authors' own graph.

Simple juxtaposition of examples (Type 1.1: juxtaposition) could be particularly prevalent for a variety of possible reasons (see Figure 2). Firstly, these are tasks intended to make students describe places, regions or states, as shown in the following example: "Compare the structural data of Germany and Nunavut (table)" (Bette at al., 2017, p. 37). This task provides comparison variables (structural data) and units (Germany and Nunavut). Comparing is not an objective in itself as it also serves the purpose of learning about a geographical situation or reproducing information from the textbook. Closed or reproductive tasks are supposed to let students access to a preconstituted knowledge-base (Osborne, 2014, p.580) and indicate a subject-centred textbook design (Lee and Catling, 2017, p.345). The fact that they were prevalent in our study is in line with previous findings that also showed this predominance of closed tasks in geography textbooks (Graves and Murphy, 2000), including textbooks with enquiry-based approaches (Lee and Catling, 2017, p. 352).

Furthermore, these very frequently set tasks have similar characteristics to everyday comparisons, and can raise students' interest as they compare something familiar (in our last example, Germany) with something unfamiliar (in our last example, Nunavut). This has been debated, Young (2009) argues that everyday knowledge has limits and that school knowledge allows more generalisations and access to “powerful knowledge”. However, using everyday knowledge can be valuable. Indeed, students are more interested and engaged in learning when local experiences and global or extralocal perspectives are connected (Klein, 1995, p. 365; Atherton quoted in Roberts, 2014, p. 193). Appealing to students’ experiences is also a way to link everyday knowledge and school knowledge and to help students acquire skills and understand concepts or generalisations (Roberts, 2014, p. 192). This method is used in enquiry-based learning approaches (present in our sample via three English textbooks).

However, as seen previously (Part I), everyday comparison is not seen here as a method used to acquire specific competencies. Tasks were classified as complex if they explicitly required explanation or reflection, or if students could choose variables or units in a reflective way. As this was not the case in our last example, we classified it into the simple category. Yet, depending on the students and their level of autonomy, or on the teacher, this type of task could also become complex. This is because “to compare” as a command verb is vague and does not systematically imply more than a simple juxtaposition: its interpretation can vary (e.g. Ministerium für Schule und Weiterbildung des Landes Nordrhein-Westfalen, 2015, p.2). Finally, it is also possible that textbook authors do not consider comparison as a competency to be developed, or do not include reflection on the comparison as a specific method. Therefore, a very large number of comparison tasks simply describe or approach comparison through a single element of the aforementioned process (Wilcke and Budke, 2019: juxtaposition (step 4)). Comparison, as discussed here, is a neutral zero-sum activity that does not participate in method acquisition and, most notably, reflection on the mechanism of comparison is absent or assumed to be self-evident.

Nomothetic and idiographic processes (see Part I) were present in many of the complex tasks studied. Consequently, there was a high concentration of comparison tasks based on these purposes (see Figure 2). This confirms the importance of comparison in geography education to gain knowledge through an inductive process as stressed in geography education research (Köck and Stonjek, 2005; Laske, 2013), in French curricula (Eduscol, 2016, p.4; Le Mercier, 2010, p.66), as it is also used in scientific comparison (Lijphart, 1971). An example of a nomothetic-oriented task is: “How do emerging countries differ from developed and less developed countries?” (Janin et al., 2019a, Janin et al., 2019b, p. 125). In this example students are asked, using a map and a table presenting different countries, to compare examples, to build definitions and give the general characteristics of different development levels. This kind of passage from particular examples to general rules was found to be particularly prevalent in this study. Many tasks also adopted idiographic purposes, and were dedicated to better analysing and differentiating examples in an interpretive way, such as: “The earthquakes in Haiti and at Christchurch were of a similar magnitude but the death toll in each area was completely different. Why was this?” (Skinner et al., 2016, p. 209). Here students have to process a comparative statement between two natural catastrophes and their consequences in two different locations. To fulfil the task, students need to take different factors into account and better understand each of the places while identifying the differences between them.

Overall, the results showed a focus on inductive processes for comparison tasks, in a sample where the proportion of simple and lower-order tasks was higher than expected in a competency-oriented geography curriculum.

### 4.2 Comparison task types and complexity, country by country: a few national differences

**Figure 3:**
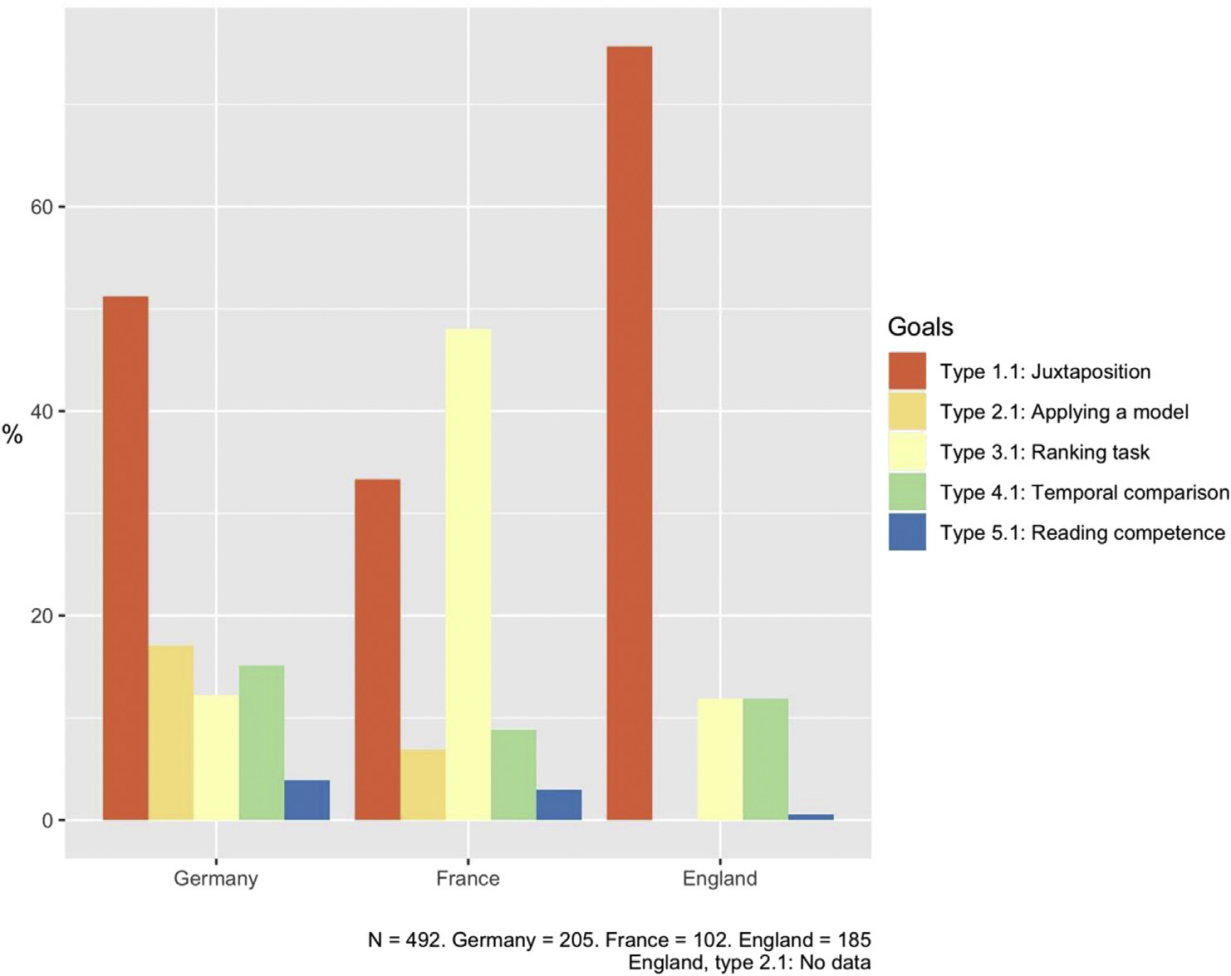
Distribution of simple goals to be achieved in comparison tasks. Authors' own graph.

**Figure 4:**
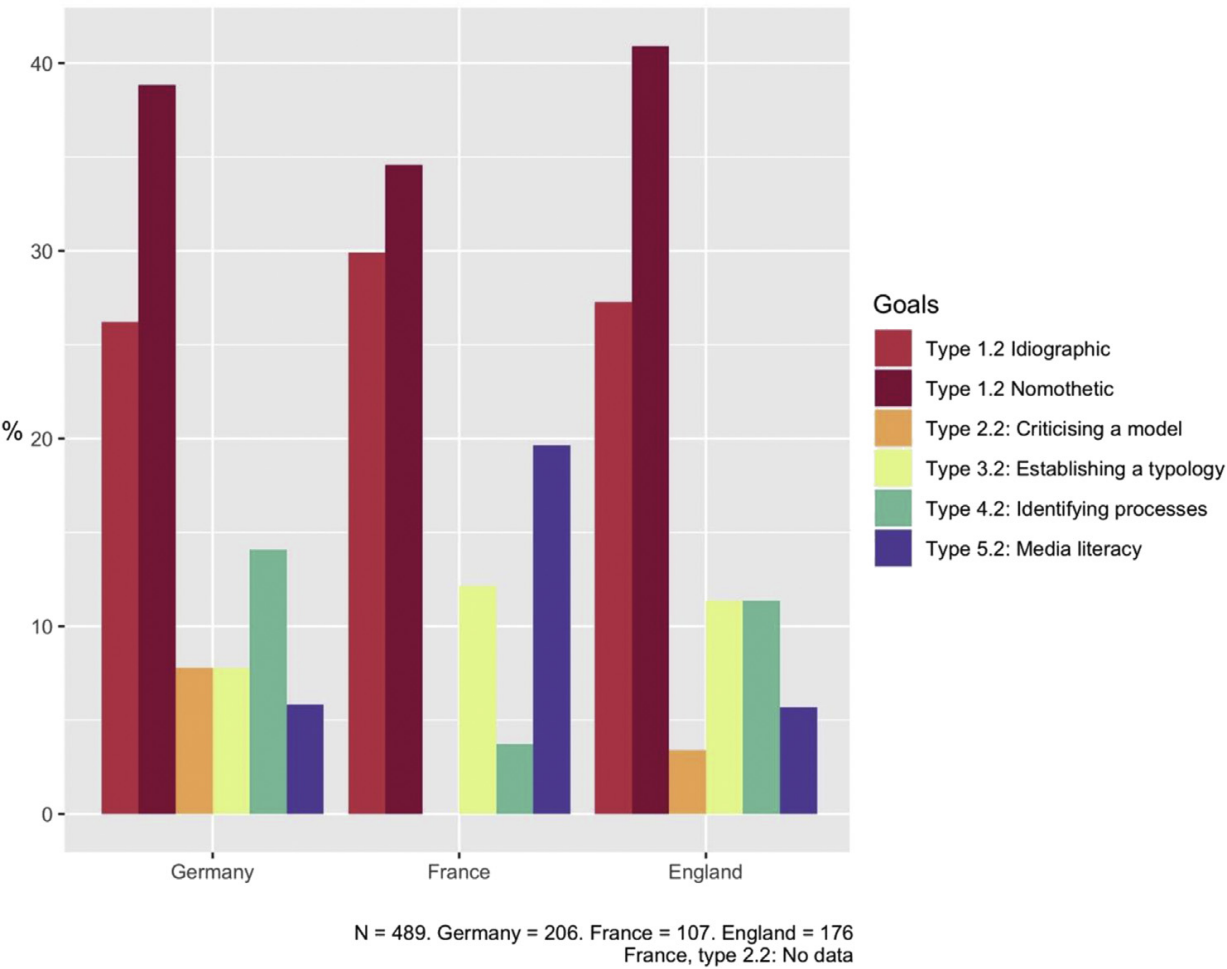
Distribution of complex goals to be achieved in comparison tasks. Authors' own graph.

After stating overall results, in the following section we present a more precise analysis of the distribution, country by country, of simple and complex tasks (respectively 492 and 489 of the total of 981 tasks, see Figures 3 and 4).

Results showed the dominance of very easy exercises aimed at simple juxtaposition, particularly in German and English textbook tasks. German textbook tasks were less polarised than the other countries with a more even distribution of 1–2 goals of comparison. Tasks aiming to apply models, rank units or make comparisons in time were substantial in German textbook tasks. However this was particularly the case in textbooks from North Rhine-Westphalia and tasks from textbooks from Berlin-Brandenburg were more polarised. In France, two types of comparison were particularly common: ranking and juxtaposition tasks, with ranking being the more predominant of the two. The following task serves as an example: “Compare Chinese investments with those of other countries. How did they increase?” (Janin et al., 2019, p. 105). Here, students use a graph to solve the task, which is located at the beginning of a case study about Chinese economic presence and spatial influence in Africa, and aims to show growing Chinese investments in Africa as compared to investments from other non-African countries. No explanation is asked for, instead ranking is used in this example as a starting point for further research questions and analyses. Finally, the focus on juxtaposition was particularly visible in English textbook tasks, while ranking and temporal comparison tasks were less evident but also common.

Studying the distribution of comparison types for complex purposes we observed the preponderance of the first types of comparison, in its nomothetic and idiographic dimensions, in all three countries (see Figure 4). Tasks with nomothetic aims were more common in all textbooks in general, although the gap between these and the idiographic aims was less marked in French textbooks. German and English textbooks had a relatively homogeneous distribution, although the German books did consistently use and critique models with a deductive approach. In this country, identifying processes also stood out, relatively speaking. For such tasks, the goal is to explain, define and characterise temporal changes such as demographic transition and economic development over time. For example, in the following task, students have to describe and explain the concept of deindustrialisation: “These images show the conversion of the “Zollverein” coal complex. It is a part of a change in the Ruhr area. Describe the two photos and compare them. Name this change and its causes” (Amstfeld et al., 2012, p.93). We also noted greater emphasis on temporal comparison to identify processes and reflection about typologies in English textbooks. French textbooks differed from the other two countries in that they did not use comparison to evaluate and critique theoretical models, and the use of comparison to develop competencies in media literacy was more developed than in the other countries. This is also linked to media literacy and the analysis of documents being part of the “Baccalauréat”, for which students are taught to reflect on and critique documents. Furthermore, the curriculum intended for the oldest students included a chapter reflecting on the use of maps, entitled “Maps to understand the world”. As an example, in the following task, students have to compare two maps; one of global demographic growth and another of global access to water. Students are asked to write an essay and analyse both documents. Different instructions guide them in this task, including questions such as: “Do the documents provide two similar, complementary or different views of the world? Justify your answer.” (Janin, 2016, p. 55). Here students have to reflect on the documents provided as sources and train their media literacy.

In general, our analysis demonstrates how comparison tasks aim towards slightly different goals in the three countries, even if common trends are observable in the overall distribution with a focus on juxtaposition for simple tasks, and on idiographic or nomothetic objectives for complex tasks.

### 4.3 The integration of comparison tasks in textbooks: trends towards different purposes in the countries studied

How are comparison tasks integrated into different parts of the textbook chapters? We analysed this question by taking into account the location of the tasks within a chapter using qualitative content analysis.

**Figure 5:**
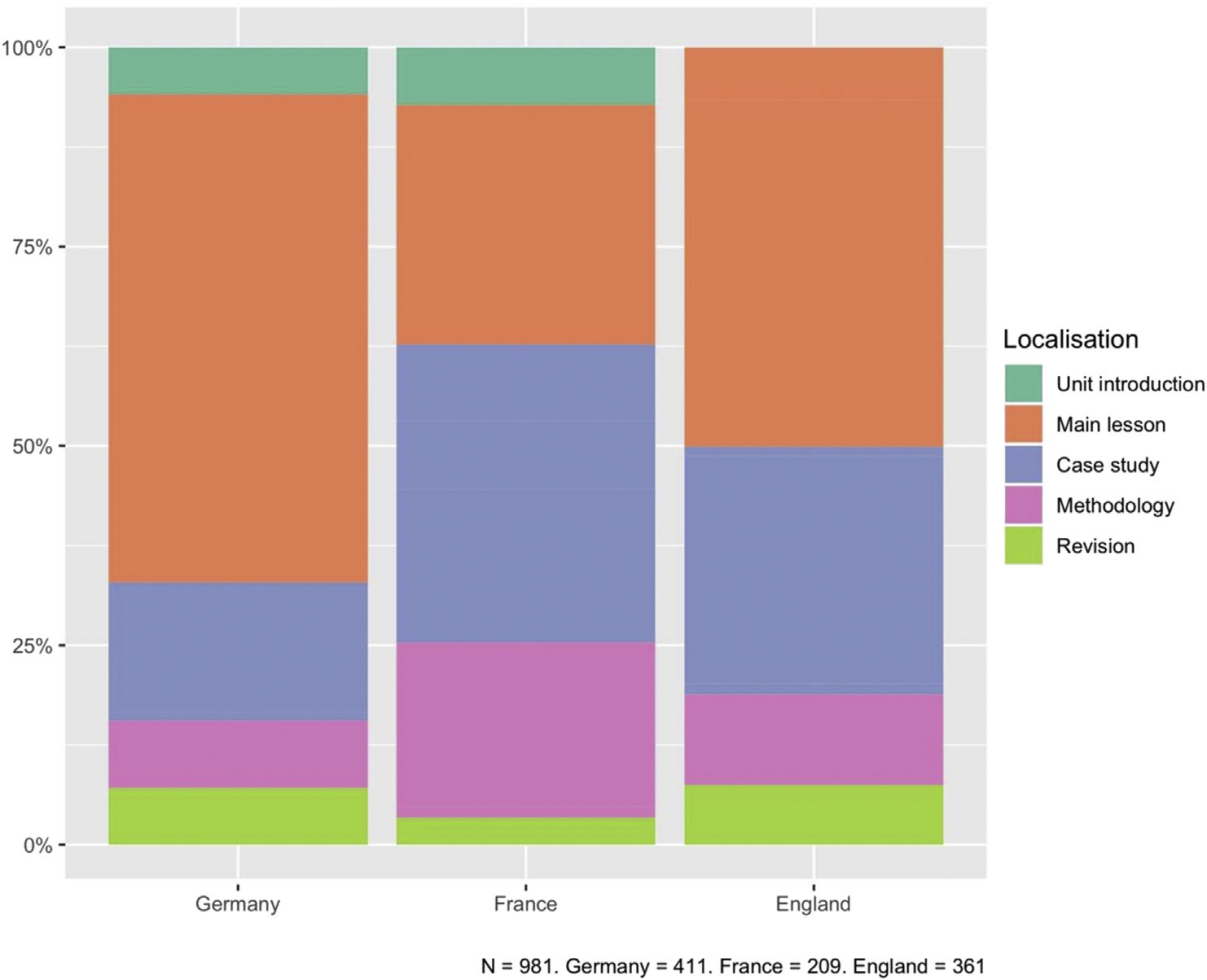
Location of the comparison tasks in the teaching unit. Authors' own graph.

Firstly, the distribution of comparison tasks in the different parts of the textbook chapters varied between the countries studied (see Figure 5). While German curricula were thematic and did not make particular use of case studies, in France and England case studies played a more important role and therefore entailed more comparison tasks. In French textbooks, there were also more comparison tasks in case studies than in the main lesson.

This specific structure of French textbook chapters, where case studies are used to arrive at general concepts and rules through an inductive process (Eduscol, 2016, p.4; Le Mercier, 2010, p.66), was reflected in the characteristics of the comparison tasks. The results showed a higher proportion of comparisons that contained an interplay between case studies and other scalar levels: while 12.91% of the comparison exercises had a scalar dimension in France, only 4.13% and 4.15% did in Germany and England, respectively. This scalar dimension was used, for example, in specific case studies to compare processes (for example, demographic change or migration) in France with the same processes on larger scales, or globally.

German and French textbooks used comparison in introductions, while English books did not. Comparison tasks in introductions were mainly either inspired from everyday comparison approaches and aimed to relate the material to students' experiences or representations, or serve the purpose of stating working hypotheses or problems in a scientific way. This can be seen in the following task, which introduces the chapter “Urban areas and their inhabitants”, and where two images of two cities must be compared: “Write down the similarities and differences between the two spaces presented. Develop a hypothesis to answer the following question: what are the visible functions of these landscapes?” (Plaza et al., 2016, p. 162–163). Through this task, students are asked to identify common elements in the definition of urban areas; in the images provided with the task, urban areas appear as economic centres, as spaces where there are transportation and mobilities, that are home to thousands of inhabitants, but they also seem different in terms of living conditions and basic equipment. The task serves to introduce these elements as working hypotheses that are examined in the chapter's subsequent pages. Working with students' representations, everyday life and working with hypotheses are some of the different tools that can be used to initiate a stimulus and “create a need to know” (Roberts, 2003, p. 44) in enquiry-based learning approaches. These tools are important to raise students' interest and improve their learning (Ferretti, 2013, p. 104). In the three countries, textbooks appealed to students' experiences via case studies or tasks, or via fieldwork, particularly in England, as enquiry-based approaches are supposed to do (Roberts, 2014, p. 204; Klein, 1995, p. 365).

Lastly, there was a greater emphasis on comparison in methodology pages in France, although for the three countries comparison tasks were included in these sections. In the methodology pages comparison was used to practice other skills, such as media literacy or mapping competencies. In the revision pages the goal was to apply knowledge without working on specific skills related to comparison (as in the example from Janin, 2016, p.55, see part C) or to react to an existing comparison in order to evaluate it. Consequently, we found that comparison tasks were used differently in textbooks from each of the three countries studied, and served different goals depending on their location within the textbook.

To confirm whether or not there was a link between a task's location within a textbook and its purpose, we conducted a correlation test using Pearson's Chi-Square independence test. The following cross-tabulation was obtained (see Table 3):

**Table 3:** Results from the Pearson's Chi-squared test of independence between a task's location and its type (See Part I). This contingency table presents the observed totals, [the expected cell totals], (the percentages per row).

Location in the chapter	Type 1	Type 2	Type 3	Type 4	Type 5	*Total*
Lesson	339 [328.31]	45 [34.90]	77 [79.08]	58 [62.72]	16 [29.99]	*535 (54.54%)*
Case study	174 [160.17]	7 [17.03]	46 [38.58]	32 [30.60]	2 [14.63]	*261 (26.61%)*
Methodology	53 [74.87]	6 [7.96]	16 [18.03]	18 [14.30]	29 [6.84]	*122 (12.43%)*
Revision	36 [38.66]	6 [4.11]	6 [9.31]	7 [7.39]	8 [3.53]	*63 (6.42%)*
*Total*	*602*	*64*	145	115	55	*981 (100%)*
*X*^*2*^ ​(12, ​*N* ​= ​981) = 117.457, ​*p* < 2.2e-16						

Our results showed that there was a significant relationship between the variables. Lessons and case studies tended to involve more inductive (Type 1) tasks, as expected, but involved fewer tasks enhancing media literacy (Type 5). Tasks located in methodology and revision sections tended to include more comparison enhancing media literacy (Type 5). Additionally, case studies tended to involve fewer deductive processes (Type 2), as expected. This result confirmed the aforementioned inductive function of case studies.

We also confirmed national teaching trends towards induction or deduction by locating the different comparison types in textbook sections, country by country: French textbooks tended to use inductive comparison tasks (Type 1) in case study sections, with 44.66% of Type 1 tasks in French textbooks located in case studies, whereas for German textbooks the same was only the case for 20.92% of the Type 1 tasks, and for English textbooks, the figure was 30%. On the other hand, in German textbooks, 80.39% of deductive comparison tasks (Type 2), which we already identified as more common than in the two other countries (see heading B. in this section), were concentrated in the main lesson, for England the figure was 50% and France only 7%. These results enabled us to refine our previous results and showed a slight difference in teaching orientations between countries, with German textbooks using predominantly deductive processes and French textbooks using predominantly inductive processes. In comparison, English textbooks tended to take the middle road, with no specific location for Type 1 or Type 2 processes. The specific focus on “enquiry-based learning” in the English textbooks included in our sample, therefore, did not clearly stand out.

### 4.4 Task complexity variation in relation to location and students’ age: discontinuous comparative competency building

How does task complexity vary? Can we assess variations in relation to the location of tasks? And in relation to the age of students? To answer these questions, we first tested the correlation between tasks’ locations and their complexity (see Table 4):

**Table 4:** Results from the Pearson's Chi-squared test of independence between a task's location and its complexity. This contingency table presents the observed totals, [the expected cell totals], (the percentages per row).

Location in the chapter	Complex	Simple	*Total*
Lesson	246 [266.68]	289 [268.32]	*535 (54.54%)*
Case study	137 [130.10]	124 [130.90]	*261 (26.61%)*
Methodology	73 [60.81]	49 [61.19]	*122 (12.43%)*
Revision	33 [31.40]	30 [31.60]	*63 (6.42%)*
*Total*	*489*	*492*	*981 (100%)*
*X*^*2*^ ​(3, ​*N* = 981) = 8.9587, ​*p* = .02985			

Our results showed that there was a significant dependence between variables. Tasks located in the main lesson tended to be simpler than expected, whereas tasks located in case studies, the methodology and the revision sections of textbooks were more complex than expected. A variety of different reasons may explain these results. Firstly, lesson sections entail fewer complex tasks than simple tasks because, for the textbook authors designing the section, lessons traditionally aim to provide knowledge that can be effectively taught through data comparison. The limited extent to which topics are presented in textbooks often does not allow for more complex comparisons, where different materials and comparison variables have to be considered. Consequently, complex tasks requiring more time and reflection are often located in other sections of textbooks. Yet, knowledge building and methodology competencies should not be systematically differentiated; meaningful tasks should aim to develop comparative competencies through reflection and argumentation on the comparative process at the same time as helping students acquire geographical knowledge and judgement and communication competencies.

Textbooks also integrated tasks to a different extent depending on the age of students. We analysed this via a Pearson's Chi-Square test and investigated the relationship between the complexity of comparison's objectives and the age of students (see Table 5):

**Table 5:** Results from Pearson's Chi-Squared test of independence between task complexity and the age of students for which textbooks were designed. This contingency table presents the observed totals, [the expected cell totals], (the percentages per row).

Age of students	Complex	Simple	*Total*
10–11	63 [74.27]	86 [74.73]	*149 (15.19%)*
12–13	79 [94.71]	111 [95.29]	*190 (19.37%)*
14–15	160 [150.54]	142 [151.46]	*302 (30.78%)*
16 +	187 [169.48]	153 [170.52]	*340 (34.66%)*
*Total (981)*	*489*	*492*	*981 (100%)*
*X*^*2*^ ​(3, ​*N* = 981) = 13.404, ​*p* = .003			

Firstly, the proportion of comparison tasks increased with the age of the students for which the textbook was designed. Task complexity also tended to increase with the age of students and, as such, the two variables were not independent. These results were significant and revealed that textbook authors linked comparison with higher competencies, escalating with the students’ age. Furthermore, we examined the distribution of complex tasks into comparison types in the different age levels per country. In all group levels Type 1 tasks (nomothetic or idiographic processes) were predominant. Type 4 tasks were frequent in lower age groups (temporal comparisons: 20.63% of tasks). In older groups, the distribution between the various types of tasks was less polarised, although the use of models (Type 2: 8.55% of tasks intended for 16+ year-old students, especially for Germany were they made up 12.94% of tasks) and document critiques (Type 5: 13.35% of tasks, and higher in France with 34% and England with 13.46% of tasks) stand out. As seen before (see Part I), comparison is a specific process that has to be developed and reflected upon, and increasing task difficulty seems appropriate in order to build this competency during secondary school according to a constructivist approach. Also, teaching students to carry out complex types of comparison is more likely to help students acquire and develop methodological and geographical competencies. It is also meaningful in the sense that reflective comparison can help develop critical thinking and judgement. However, younger students are also capable of complex operations, so instead of being offered too many simple and meaningless comparisons they should be given the opportunity, via a differentiated assessment, to build comparative skills progressively. It should also be possible in lower age groups to study all different comparison types without only focusing on inductive or temporal processes, which are important but do not represent the variety of comparison possibilities.

## 5 Conclusion

While comparison is a process that is presented as self-evident in curricula, and is frequently used in geography classes, previous research has, thus far, not focused on the implementation of comparison tasks in textbooks. Yet, comparison in geography is very important in scientific analysis and has been fruitful in many ways, particularly with regards to methodological and epistemological debates in the discipline (Gervais-Lambony, 1994; Nijman, 2015; Brenner and Keil, 2006). Our study analysed the extent to which geography textbooks include comparative tasks that foster such geographical competency. To better categorise comparison tasks according to scientific objectives, we built a typology of comparison tasks in textbooks, the validity of which was subsequently tested. We used this classification for our international study, which compared geography textbooks from Germany, England and France, and allowed an initial exploratory approach to comparative tasks through qualitative and quantitative analysis.

Our empirical results showed that tasks tended to focus on only one type of comparison (Type 1) in the three countries studied. This can be explained by the fact that Type 1 objectives are fundamental objectives in geography (Peck, 2015; Robinson, 2011). They are then, as we might expect, practised as such in secondary education, however, too often using lower-order tasks involving juxtaposition (Type 1.1). It is also regrettable that we found no greater variability in objectives of comparison between the tasks. The ‘divide’ between school- and university-based geographies (Firth, 2011, p. 158; Roberts, 2014, p. 201–202; Clerc, 1993, p. 114), could be a reason why textbook authors tend to favour Type 1 and do not sufficiently consider other possible types used in scientific geography. However, comparison processes, such as the elaboration of typologies (Type 3), criticism of sources (Type 5), modelling or questioning of models (Type 2), and identifying the processes leading to the constitution of spaces or places (Type 4), are part of a geographer's toolbox and should be taught in secondary education via relevant tasks.

It is regrettable that many comparison tasks we analysed did not promote the development of complex reasoning or argumentation. Tasks were often exclusively oriented towards the acquisition of simple knowledge, with a focus on closed teaching approaches observable in textbooks and curricula, as other research findings in geography education have already showed in the three countries (Budke, 2011; Lee and Catling, 2017; Colin et al., 2019). A large proportion of comparison tasks had only simple cognitive objectives and were not oriented towards competency acquisition. This result may suggest a lack of reflection on the part of textbook authors about the specific task and process that comparison constitutes (Wilcke and Budke, 2019). It also reflects the common misconception that the content of geography textbooks allows direct access to a “geographical reality”, and therefore students do not need to produce knowledge or acquire complex skills (Tutiaux-Guillon and Nicole, 2008, p. 119). Including meaningful comparison tasks in textbooks could reverse this situation: research shows that tasks allow students to learn specific ways of processing information, and not only to reflect on content (Doyle, 1983, p. 161). Moreover, in order to be able to critique or debate on geographical content, students must have access to the “epistemic tools provided by the discipline to construct knowledge” to acquire not only procedural knowledge, but also “knowledge on their own knowledge”, and therefore “powerful knowledge” (Young, 2014, p.20; Maude, 2016, p. 75).

Our typology appears to be a robust tool to analyse and develop tasks for several reasons. Firstly, the types obtained through category-building corresponded to different types of scientific comparison and types of tasks attested by scientific research (Lijphart, 1971; Krathwohl, 2002; Jo and Bednarz, 2009). Secondly, the reliability of our typology was checked through its use on our sample by different judges and the calculation of interrater agreement allowing a triangulation by researcher (Miles et al., 2014, p. 299). The validity of the typology was also confirmed by the fact that all comparison tasks could be classified within it. Lastly, the results of our quantitative analysis, carried out on a large and diverse sample, confirmed prior research results showing textbooks’ focus on lower-order and closed tasks (Budke, 2011; Lee and Catling, 2017; Colin et al., 2019), suggesting that the typology should be transferable to other contexts (Miles et al., 2014, p. 314).

Our international analysis showed common shortfalls across the different countries, even in the English textbooks using “enquiry-based” approaches. It also showed differences between the countries studied in curricular approaches as well as textbook design. Although our study did not analyse textbooks from different publishers within the countries, our results are in line with what research has described as different subject cultures (Hericks and Körber, 2007, p.31–32). Much could be learned from each specific textbook culture and applied in the different countries. For example, the use of case studies and fieldwork, particularly prevalent in France and England, is useful for implementing comparison tasks and methods, especially due to its inherent scalar dimension which is an important aspect of geographical thinking (Colin et al., 2019, p. 6). The systematic use of command verbs in task formulation in Germany is also useful for promoting the development of comparison competencies. The media literacy and documents critique tasks in French textbooks are also crucial to the development of reflection and argumentation on geographical content.

Our typology, specific to comparison tasks in geography education, constitutes an analytical framework that can be used to help textbook authors and teachers identify and develop meaningful tasks to grow students' skills regarding comparison. It can particularly help in the analysis and design of tasks enhancing complex reasoning and argumentation. Varying the exercises and providing more space to research situations would appear to be more effective in a competency-oriented geography class, where comparison would be a tool used deliberately and in an informed manner. Further research could focus on developing a model to enhance this “comparative competency” as a goal in itself, indispensable to solid knowledge building and critical thinking.

## Declarations

### Author contribution statement

Marine Simon: Conceived and designed the experiments; Performed the experiments; Analyzed and interpreted the data; Contributed reagents, materials, analysis tools or data; Wrote the paper.

Alexandra Budke: Conceived and designed the experiments; Analyzed and interpreted the data; Wrote the paper.

Frank Schäbitz: Conceived and designed the experiments.

### Funding statement

This work was supported by 10.13039/501100001659Deutsche Forschungsgemeinschaft [57444011 - SFB 806].

### Competing interest statement

The authors declare no conflict of interest.

### Additional information

No additional information is available for this paper.

## Footnotes

1 In French: “*consigne”*.
